# Notes and News

**Published:** 1897-10-16

**Authors:** 


### Notes and News.

(Collected and Communicated.)

The site has been fixed and the plans passed of the new Victoria Hospital at Kingston-on-Thames. Upwards of £3,300 has been collected, and annual subscriptions amounting to £276 are already promised.

A Palestine exhibition is being held during the present week at the Hampstead Vestry Hall, Haverstock Hill, the proceeds of which are to promote the building of new hospitals in connection with the Society for Promoting Christianity.

The new medical block in connection with the Aberdeen Royal Infirmary is now nearly completed. It has been erected in commemoration of the Queen's Jubilee. The building is four storeys high, and is in the same style of architecture as the rest of the infirmary.

Mr. W. Tone, the honorary treasurer of the Sunderland Blind Asylum, has purchased a block of buildings adjoining the institution, and presented it to the authorities, to secure the extension of the asylum which is desired.

The subscriptions for the erection of the new fever hospital at Sydney have reached upwards of £15,000. The institution is to be called the Queen's Memorial Fever Hospital, and it is intended, as its name indicates, to commemorate the Queen's Jubilee.

A proposal has been made to utilise gas street lamps for a public water supply. The hot water would be procured through the penny slot system, and the satisfactory adjustment of this system to the case has been demonstrated. The sanitary control and inspection of the apparatus has to be carefully considered, and this might prove a large and difficult matter. If the water is to be used for drinking purposes, the scheme, which is undoubtedly a convenient one, might prove a new source of danger to public health.

In Paris a list of doctors ready to attend in case of emergencies occurring at night is published for the convenience of the public. Originally, we learn, a fee of 10 francs was the standard payment, but more recently a pool has been instituted, and the result divided quarterly amongst the doctors. This system has alienated the better class practitioner, and now the employment of the whole class has become endangered by the death of a patient treated by one of the members who lives on £15 per annum, with a stock of instruments as scanty as his income.

The international conference which was opened on October 11 will consider the questions: "What justification is there for regarding the bacillus of leprosy as the cause of the disease?" and "In what way is the infection conveyed?" Among other introductory papers set down for reading is one by Mr. Jonathan Hutchinson, of London.

No one is surprised to learn that the death-rate during an epidemic such as that raging at Maidstone at present should exceed 30 per week; but the facts which Dr. Templeman points out in relation to child mortality in Dundee certainly appear extraordinary. During last August it is shown that no less than 130 children died in Dundee under the age of five years. The principal complaints recorded are gastric and intestinal in character.

The furnishing contracts at the Birmingham General Hospital have created quite a social storm locally. The Furnishing Committee are accused of "sweating," as it is stated that the contracts have got into the hands of firms who have sub-let to others not conducted on trade union principles. It appears that the proviso as to sub-letting appears in the specifications, although the committee exerted themselves to provide against the sub-letting of contracts.

A handsome building, to serve as a home for destitute crippled children, has been opened at Gosforth, Newcastle. It is to take the place of a less complete and smaller institution at Wallsend, and will contain 73 boys and 37 girls. The movement was originated by Miss Emma Watson, who provided originally for six little cripples at Whickham. The present building has cost £10,000, of which £8,500 has been subscribed. The largest contributors have been Mr. and Mrs. Philipson, who have given £1,100 towards the building fund, and £2,000 for the endowment of five cots.

Wherever a Hospital Saturday Fund, with its attendant tradition of street collections and demonstrations, is established, the same difficulties arise sooner or later. These difficulties are, we find, as apparent in Australia as in England. Quite recently a Sydney paper records the deadlock at a place called Balmain, where various Friendly Societies desired to organise a street collection and demonstration on behalf of the local cottage hospital. At a meeting on the subject those present were much divided in opinion, and it ended in the casting vote being given by the chairman to decide the matter.

The recently issued report of the Local Government Board contains interesting evidence of the general trustworthiness of the medical profession, as measured by the manner in which its members discharge their official duties. In the course of the year 1896 a certain number of officers under the Poor Law were either dismissed or were required to resign their appointments on account of some misconduct or neglect of duty. Among workhouse masters this fate befell one in every 96; among relieving officers, one in every 125; among chaplains, one in every 282; among medical officers, one in every 760. The average conduct of medical officers has thus been more than three times as good as that of chaplains, and with the other classes it is unnecessary to institute a comparison.

